# Supplementary material for: The Experiences of Community Health Workers in Preventing Noncommunicable Diseases in an Urban Area, the Philippines: A Qualitative Study
Source: Healthcare (Basel). 2023 Aug 30;11(17):2424. doi: 10.3390/healthcare11172424 (PMC10487527; doi:10.3390/healthcare11172424)
Supplement: Supplementary file 1 [file healthcare-11-02424-s001.zip › healthcare-2565124-supplementary.pdf]

Interview Guide for Baragay Health Workers

	Question	Probe
I.	Consciousness toward the prevention of non-communicable diseases (NCDs)	
1.	What is your understanding of the NCDs prevention?	-Please explain your opinions.<br>-Why do you think so?
2.	Are you willing to prevent NCDs in the community?	-YES<br>：Why do you think so?<br>-NO<br>：Why don't you think so?
3.	How important do you think the NCDs prevention is?	-Why do you think so?
II.	Activities and roles for the prevention of NCDs	
1.	What are activities of BHWs for the NCDs prevention in the community?	-Please explain more.<br>-Contents, frequency, duration
2.	How do you deal with the problems or concerns relate to the NCDs prevention in the community?	-Please explain more.
3.	What are the roles of BHW for the NCDs prevention in the community?	-Please explain more.<br>-Why do you think so?
III.	Difficulites that BHWs need to address for the prevention of NCDs	
1.	What are the biggest challenges for the NCDs prevention in the community for community health workers?	-Please explain more detail.<br>-Why do you think so?
2.	Please explain your opinions why individuals have difficulty in preventing NCDs in the community?	-Pleasse explain more detail.<br>-What do you need to improve the problems? Why?
